# Understanding less than nothing: children's neural response to negative numbers shifts across age and accuracy

**DOI:** 10.3389/fpsyg.2013.00584

**Published:** 2013-09-10

**Authors:** Margaret M. Gullick, George Wolford

**Affiliations:** Department of Psychological and Brain Sciences, Dartmouth CollegeHanover, NH, USA

**Keywords:** integers, negative numbers, fMRI, development, distance effect

## Abstract

We examined the brain activity underlying the development of our understanding of negative numbers, which are amounts lacking direct physical counterparts. Children performed a paired comparison task with positive and negative numbers during an fMRI session. As previously shown in adults, both pre-instruction fifth-graders and post-instruction seventh-graders demonstrated typical behavioral and neural distance effects to negative numbers, where response times and parietal and frontal activity increased as comparison distance decreased. We then determined the factors impacting the distance effect in each age group. Behaviorally, the fifth-grader distance effect for negatives was significantly predicted only by positive comparison accuracy, indicating that children who were generally better at working with numbers were better at comparing negatives. In seventh-graders, negative number comparison accuracy significantly predicted their negative number distance effect, indicating that children who were better at working with negative numbers demonstrated a more typical distance effect. Across children, as age increased, the negative number distance effect increased in the bilateral IPS and decreased frontally, indicating a frontoparietal shift consistent with previous numerical development literature. In contrast, as negative comparison task accuracy increased, the parietal distance effect increased in the left IPS and decreased in the right, possibly indicating a change from an approximate understanding of negatives' values to a more exact, precise representation (particularly supported by the left IPS) with increasing expertise. These shifts separately indicate the effects of increasing maturity generally in numeric processing and specifically in negative number understanding.

The development of numerical cognition includes a progression from an innate approximate recognition of quantity (Xu and Spelke, 2000; Lipton and Spelke, 2003; Xu et al., 2005; McCrink and Wynn, 2007) to a sensitivity to quantity manipulations and violations (Wynn, 1992; Barth et al., 2005, 2006, 2008; Gilmore and Spelke, 2008), an understanding of precise values and the symbols which represent them (Ansari et al., 2005; Gilmore et al., 2007; Roux et al., 2008; Holloway and Ansari, 2009; Lyons and Ansari, 2009; Cantlon et al., 2009), and eventually the ability to perform symbolic arithmetic (Menon et al., 2000; Rivera et al., 2005) and a knowledge of higher-order mathematics (Anderson et al., 2011). These steps reflect increasing expertise with tangible quantities, or symbols representing such concrete amounts. In contrast, understanding negative numbers involves conceptualizing and manipulating abstract quantities that are worth less than nothing yet have value, requiring acceptance of amounts lacking direct physical counterparts. While we have gained mastery of these numbers as adults, negatives remain difficult (Gullick and Wolford, 2013), and the process of conceptual acquisition is unclear. Learning how we come to understand this concept, and whether the brain regions supporting their understanding are similar to or different from those for easier positive numbers, can inform our knowledge of how we come to understand similarly difficult abstract ideas in both mathematics and other domains, and may be able to eventually inform educational practice and strategies.

Research has recently begun to investigate the behavioral and neural processes supporting our understanding of negative numbers. Studies with adults have indicated that we may have quantitative representations of negatives similar to those for positives (Fischer, 2003; Ganor-Stern and Tzelgov, 2008; Tsang and Schwartz, 2009; Tzelgov et al., 2009; Varma and Schwartz, 2011), and may draw on neural primary number areas for negatives, even if in a slightly less precise or mature manner (Blair et al., 2012; Chassy and Grodd, 2012; Gullick et al., 2012). However, adults have had years of practice and experience with negative numbers and much time to build these positive-like representations. No work has so far described the brain activity underlying children's use of negatives, either before or soon after school instruction on the topic.

Negative numbers are usually introduced into the mathematics curriculum at some point between fourth (Varma and Schwartz, 2011) and sixth (Education, 2010) grade, after years of instruction on and practice with positive numbers. While limited research has explored children's processing of positive numbers, even less has aimed to examine their negative number understanding. A few works have qualitatively described children's informal verbal explanations of negative numbers, which demonstrate the counterintuitive nature of these items. Negatives seem to be difficult to learn (Streefland, 1996): upon introduction, students may ignore signs (Vlassis, 2004), may inappropriately apply signs (Davis and Maher, 1993), and may order the negative end of the number line backwards (Widjaja et al., 2011). Borba and Nunes (1999) determined that while children had some ability to use negative numbers after a short introduction to the multiple meanings of the minus sign, their explanations and demarcations were idiosyncratic and most were unable to use manipulable materials in their explanations, preferring only oral descriptions. After two years of instruction, though, Varma and Schwartz (2011) demonstrated that sixth-grade children showed behavioral effects similar to those for adults for negative number comparisons. As such, children may not initially understand negative numbers, but come to use them fluently after some practice and experience. We here specifically investigate the neural systems supporting negative number use, compared to those involved in positive number processing, in pre- and post-instruction children in an effort to better understand the trajectory of the acquisition of negative number knowledge from initial processing strategies to eventual adult expertise.

Most often, investigations of adult negative number processing have examined the presence and direction of the distance effect, a typical positive-number processing result. First described by Moyer and Landauer (1967), the distance effect describes an inverse relationship between comparator distance and reaction times: comparisons involving numbers that are further apart (at a greater distance) are responded to faster and more accurately than those with numbers that are closer together. This effect has been taken to reflect the possible representational overlap between neighboring numbers on a mental number line, as ordering and choosing the greater of two numbers becomes more difficult to resolve if the items are closer together (Van Opstal et al., 2008; Holloway and Ansari, 2010; Holloway et al., 2010). Neurally, the distance effect is reflected in gradations of IPS activity: close-distance numeric comparisons elicit more IPS activity than far comparisons (Pinel et al., 2001; Ansari et al., 2006), possibly reflecting the increased effort and neural activity needed to resolve the more difficult closer comparisons.

The distance effect is conserved in children, though there are developmental changes in scale and localization. Behaviorally, while overall response times are slowed, children still demonstrate faster and more accurate responses to farther than closer comparisons (Holloway and Ansari, 2008). The size of the effect also decreases with age, as children show a greater distance effect than adults (Holloway and Ansari, 2008), and may also be related to mathematics achievement, with the effect becoming smaller and less dramatic with increasing math skill (De Smedt et al., 2009; Holloway and Ansari, 2009; but see Schneider et al., 2009).

Like adults, children may also demonstrate a significant effect of distance neurally, but the pattern of responses differs by age. The IPS demonstrates a distance effect to non-symbolic numbers from at least age four (in an adaptation paradigm; see Cantlon et al., 2006) or age six or seven (in a comparison paradigm; see Ansari and Dhital, 2006; Cantlon et al., 2009). Comparison effects for symbolic numbers, though, may not be IPS-based. Instead, children (ages 8–12) may show only a frontal distance effect, particularly in the dorsolateral prefrontal cortex and inferior frontal gyrus. The IPS does demonstrate significant activity during comparisons, but is not distance modulated (Ansari et al., 2005). Temple and Posner (1998) did note a parietal distance effect for both symbolic and non-symbolic comparisons in five-year-olds using event-related potentials, indicating that parietal number areas may be responsive to symbolic numbers even from a young age, but the spatial resolution of ERPs makes source localization difficult. At some point, the distance effect may shift to be both frontal and parietal, then to the adult parietal-only effect, but the trajectory of these changes has not been fully described. Generally, though, these shifts are consistent with the developmental finding wherein activity related to mathematics processing shifts posteriorly with age (Rivera et al., 2005).

Behaviorally, several studies have tested adult responses to negative numbers, most often in paired comparison paradigms. Negative pairs have consistently demonstrated a typical distance effect in both simultaneous (Varma et al., 2007; Tzelgov et al., 2009; Varma and Schwartz, 2011) and sequential (Ganor-Stern et al., 2010) single-digit negative number paired comparisons. Recently, Gullick, Wolford, and Temple (Gullick et al., 2012) also tested the adult brain activity supporting paired comparisons with positive and with negative numbers. First, across comparison distances, negative numbers showed increased parietal-lobe activity, including in the IPS, relative to positive pairs, along with increased caudate and decreased frontal-lobe activity. This overall parietal increase may be due to differences in difficulty between the signs (Gobel et al., 2004; Blair et al., 2012; Chassy and Grodd, 2012). Importantly, though, Gullick et al. (2012) found that negatives also showed typical distance-modulated responses both behaviorally and neurally, including in the IPS, which was also greater for negative than for positive pairs in each case; positive comparisons again demonstrated a stronger effect of distance frontally than did negatives. This difference indicates that while processing abstract negative numbers strongly engages primary number areas, it may less draw on frontal secondary regions than concrete positive numbers. As a greater distance effect has been taken to indicate a less mature representation of number (De Smedt et al., 2009; Holloway and Ansari, 2009), the representation of negative numbers was proposed to be less precise than that for positives, leading to this more dramatic distance effect.

Adults may thus understand negative numbers as individual quantities arranged along the leftward end of a bidirectional number line. These representations seem to be supported by similar activity in the same quantity-sensitive regions as positive numbers. This mature usage, though, may stem from years of experience and practice with negatives. How do children, who have little or no formal experience with these concepts, respond to negative numbers?

In summary, numeric negativity appears to be a difficult concept to acquire. Children may be able to use negatives in some limited situations even before instruction, but such knowledge is typically limited to informal situations (Mukhopadhyay et al., 1990) and is unstable (Borba and Nunes, 1999). After some instruction, children may demonstrate a typical behavioral distance effect to negative comparisons, (Varma and Schwartz, 2011), but whether children also show a typical neural distance effect for negatives, or after what amount of practice such an effect may appear, is not known.

We here aimed to examine whether children showed a neural distance effect for negative number comparisons similar to that for positive numbers. In our area (New Hampshire and Vermont), negative numbers are formally introduced in the sixth grade (2010). As such, fifth-graders were used as a pre-instruction group, and seventh-graders as a post-instruction cohort. Fifth-graders were not expected to be completely naïve to negatives, so should be able to perform simple comparisons but not able to use negatives in arithmetic or more complex situations. Seventh-graders, who should have at least one year of formal experience with negatives, were expected to show a greater proficiency with negatives, including in comparisons.

A distance effect for positive number comparisons was expected in certain neural locations within each age group. In line with Ansari et al. (2005), the ten- to eleven-year-old fifth-graders were expected to demonstrate a frontal distance effect for positive-number comparisons. The 12- to 13-year-old seventh-graders were at or beyond the upper limit of Ansari et al. (2005), and could show both frontal and parietal distance effects for positive number comparisons. The presence, direction, and location of a negative number comparison distance effect was then explored within each age group. This method allows determination of whether effects “match” across signs, even as the effect location shifts between age groups, and thus whether negative numbers are processed using the same neural mechanisms as positives at each instructional stage (e.g., whether negative comparisons evoke the same frontal or parietal distance effect as positive pairs).

## Methods

### Participants

Participants were 16 (6F) fifth-graders, ages 9;11–11;9 (mean = 10;8 years), and 15 (5F) seventh-graders, ages 11;9–13;5 (mean = 12;8 years; see Table 1 for demographic information). All were right-handed, as assessed by the Edinburgh Handedness Inventory (Oldfield, 1971), with no history of learning disorder or neurological damage. Nine additional participations were excluded, 4 due to artifact from braces, 2 due to response recording problems, 2 due to excessive movement, and 1 due to use of drugs affecting white matter integrity. Participants were paid $25 and given several small prizes.

**Table 1:** **Demographic characteristic of each age group**.

	**Fifth-graders**	**Seventh-graders**	
N	16	15	
Age	10;8 (7.66 months)	12;8 (5.89 months)	*p* < 0.001
Age range	9;11–11;9	11;9–13;5	
KTEA-II Math Concepts and Applications standard score	114.06 (17.48)	120.73 (9.71)	*p* > 0.2
Integer Knowledge Test (out of 62)	36.81 (7.34)	51 (7.55)	*p* < 0.001
Stroop interference standard score	52 (6.53) (14 participants)	50.47 (9.01)	*p* > 0.6

### Stimuli

Stimuli were the same as used by Gullick et al. (2012) with adults. Briefly, stimuli used pairs of numbers from −20 to 20, excluding zero. Comparison pairs were created in three main sign categories (20 positive, 20 negative, and 60 mixed comparisons), for a total of 80 unique pairs (see Table 2 for example pairs from each included category; we here focus on only positive and only negative comparison pairs, and so mixed pairs are not further discussed). Half the comparisons in each sign type were closer in distance (between 1 and 8) and used two single-digit comparators, while half were farther apart (between 12 and 19) and used one single- and one double-digit comparator. Positive and negative number comparisons used the same digits, but different signs.

**Table 2:** **Example stimuli**.

**Distance groupings:**	**Sign category**	
	**Positive**	**Negative**
Closer (distances 1–8)	3 <> 5	−3 <> −5
Farther (distances 12–19)	3 <> 16	−3 <> −16

Half the comparisons involved two presented digits, and half used thermometers. While fifth-graders were not expected to have formal experience with negatives as abstract digits, they could recognize negatives as representing very cold temperatures, given their geographic location. As such, comparisons were presented in half the runs as digits, and in half as temperatures on a thermometer. Thermometers were created using a blank canonical shape, with unlabeled side tics and red filling up to half-height. Temperature was labeled in red to the left of the thermometer by the middle tic. These thermometers were meant to invoke and reinforce the context of a temperature comparison, but not to test thermometer-reading skill. To keep participants from ignoring the numbers presented and simply visually comparing the amount of “red stuff” (mercury) in each thermometer, filling height and digit position were kept constant, making it impossible to base the comparison off area or relative vertical number-line position. As this comparison, and indeed the range of temperatures presented, was possible in either Celsius or Fahrenheit, no specific scale was given for the thermometers: participants were simply instructed to think of the numbers as temperatures and choose the warmer (or colder) temperature. We here focus on responses collapsed across presentation format.

Baseline control trials presenting a blank screen were also used.

### Procedure

After obtaining informed consent, participants completed a survey testing their knowledge of signed number usage and operations (see Appendix), which included questions requiring participants to order numbers, choose the larger number, and perform simple arithmetic with and complete word problems involving negative numbers. Participants were also given the Math Concepts and Applications subtest of the Kaufmann Test of Educational Achievement-II (KTEA-II; Kaufman and Kaufman, 2004), which tests the ability to apply mathematics knowledge to solve problems. A color-word Stroop test standardized for children (Golden et al., 2003) was also administered to all the seventh-graders, and 14 of the 16 fifth-graders. The Stroop test was administered in the same testing session as the fMRI scan for 20 of the included participants (9 5th graders and 13 7th graders), and in a separate second session between 2.5 and 4 months later for the remaining participants. Two fifth-graders could not return for a second testing session, and so Stroop scores are reported for only the 14 available participants. Scores were normed to age of test administration.

After a short practice session, fMRI data was acquired in four event-related functional runs. One half of the experimental session asked participants to choose the larger number (or warmer temperature), the other half asked participants to choose the smaller number (or colder temperature). Question and format order were counterbalanced across participants. Run lists consisted of 100 experimental trials, using one instance (left- or right-greater) of each unique comparison. Thirty-two baseline control trials were also included in each run (including three at the end of each run) at jittered intervals. Each run was ~5.5 min in length. Stimulus pairs were presented in pseudorandom order. Each unique comparison was thus presented in eight variations (within each context, in each question version, with the greater number on the left vs. right).

**Figure 1:**
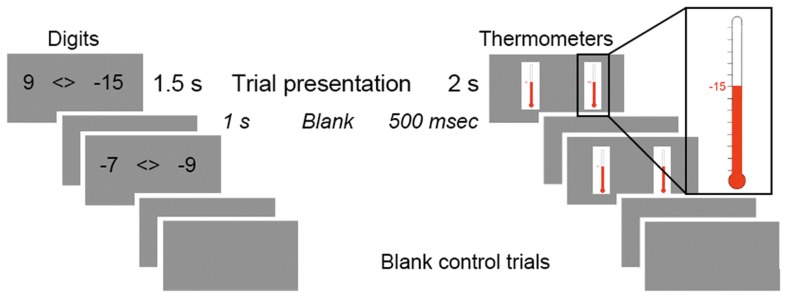
**Trial schematic.** Trials presented paired comparisons of either two digits or two thermometers. In both contexts, 1 *TR* = 2.5 s.

In each comparison trial, one item was presented on the left side of the screen, and one on the right, separated by a “<>” symbol. Digit comparison pairs were presented for 1.5 s, followed by a 1 s blank screen, and thermometer comparison pairs for 2 s, followed by a 500 ms blank screen (see Figure 1). Participants could respond at any point within the display time, but were encouraged to respond quickly and accurately. Behavioral piloting determined that these presentation periods gave participants ample time to answer on each trial while continuing to encourage speeded responses; longer presentation times for digits may have resulted in decreased task attention and less pressure to respond immediately. Stimulus presentation, trial timing, and response recording was achieved using E-Prime presentation software (Psychological Software Tools, Pittsburgh, PA). Stimulus pairs were presented using a Panasonic DT-4000U DLP projector, and each functional run was synchronized with the onset of the first trial to ensure accuracy of event timing. Response times and accuracy were measured using fiber optic button press boxes (Cedrus Lumina response pads; San Pedro, CA).

### Data acquisition

Functional images were acquired in a 3T Philips Achieva Intera MRI scanner at the Dartmouth Brain Imaging Center. In each of the four functional imaging runs, we acquired 132 whole-brain T2^*^-weighted echoplanar images (EPI). 41-slice whole-brain EPI image volumes were acquired using Philips interleaved sequence maximizing the distance between neighboring slices; here, slices were acquired in intervals of 6. The following parameters were used for acquisition: slice thickness = 3 mm, no skip; repetition time (TR) = 2.5 s; echo time (TE) = 35 ms; flip angle = 90°; matrix = 80 × 80; field of view (FOV) = 240 mm; transverse plane. Two additional volumes were discarded at the beginning of each run to allow for equilibrium effects. In addition, a high resolution, magnetization-prepared rapid-acquisition gradient echo (MPRAGE) image was acquired at the end of the session, but was not used for analysis.

### Analysis

#### Behavioral analyses

Behavioral data were analyzed to determine response accuracy and ensure task attention, as well as examine the presence and direction of a distance effect, in each age group. Comparison sign types (positive, negative) were first examined for overall response time and accuracy differences. The presence and direction of any distance effects in each sign type was examined through a series of linear regressions, performed on each participant's correct response data, to determine whether response times were significantly predicted by comparison distance. The unstandardized beta coefficient for the distance regressor for each individual was then extracted, and compared across sign types using ANOVAs, with a statistical threshold of *p* < 0.05.

#### fMRI analyses

All functional data were examined for artifact by creating signal to noise maps in MATLAB (version 7.7.0 R2008b; The MathWorks, Inc., Natick, MA) with a modified script available at http://dbic.dartmouth.edu/wiki/index.php/Noise_Detection. fMRI data were processed using SPM8 (Welcome Department of Cognitive Neurology, London, UK, http://www.fil.ion.ucl.ac.uk/spm). Preprocessing steps for each participant included the following steps. *Reorientation:* The center of each functional image was reoriented such that the origin was at the midsagittal anterior commissure. *Slice Timing Correction:* Differences in image acquisition time between slices were corrected using the first slice as reference using SPM8's Fourier phase shift interpolation. *Realignment:* Head motion was realigned to the mean image using the least-squares approach and a 6-parameter rigid-body spatial transformation. Estimation was performed at 0.9 quality, 4 mm separation, 6 mm FWHM smoothing kernel, using second degree B-Spline interpolation. Reslicing was performed using fourth degree B-Spline interpolation. The realignment parameters were examined for excessive motion (defined as >1 mm motion in any direction). Two participants were excluded for head position drift exceeding 4 mm and multiple occurrences of movement spikes exceeding 2 mm. *Smoothing:* Images were smoothed using a 6 mm FWHM Gaussian kernel.

***First-level individual statistics***. All runs from each individual were analyzed together using a mass-univariate approach based on the general linear model. Two factors were modeled: comparison context (digits, thermometers), and comparison pair sign type (positive, negative, mixed polarity sensitive, mixed polarity insensitive), along with baseline control trials and the six realignment parameters from motion correction as parameters of no interest for each run. Control trials were thus modeled explicitly (see Poline et al., 2003). Error trials were included as a condition in individual analysis. A first-order parametric modulator was also included for each comparison pair sign type, marking the distance between comparators (1–19) on each trial. A high-pass filter of 128 s was used to remove slow signal drift. Summary contrast maps were created for each individual to take to second-level group analysis. Based on the specific planned group-level tests, normalized contrasts of each experimental condition versus the modeled baseline control were performed (e.g., positive > baseline, negative > baseline, etc.). The contrast for each stimulus class' parametric modulator was also created (e.g., positive distance effect > baseline). Contrasts using this modulator determined areas of the brain whose activity changes linearly in accordance with changes in distance. Mask images for each individual were examined to ensure full brain coverage. This analysis is thus the same as that used with adults (see Gullick et al., 2012).

Data from all participants was normalized to the standard (adult) SPM8 EPI template using a trilinear interpolation, writing 3 mm^3^ voxels. While pediatric templates could be used, several factors argued against their implementation in this study. First, the children included here were easily over 7 years of age, considered to be the point when the brain reaches ~95% of its adult size (see Caviness et al., 1996): fifth- and seventh-graders can thus be considered to have nearly volumetrically-mature brains, though cortical thickness and mylenation continue to develop and change. Further, previous work has demonstrated that the differences between activity localization in 7- and 8-year-old children and adults may be negligible, given the voxel sizes and smoothing kernels used in conventional fMRI analysis (Burgund et al., 2002; Kang et al., 2003). Last, some analyses compare activation across age groups. Such contrasts are best conducted on data that has all been normalized to the same space, as warping to different templates may result in systematic misregistrations and activity mislocalizations (see page 68 in Poldrack et al., 2011).

**Figure 2:**
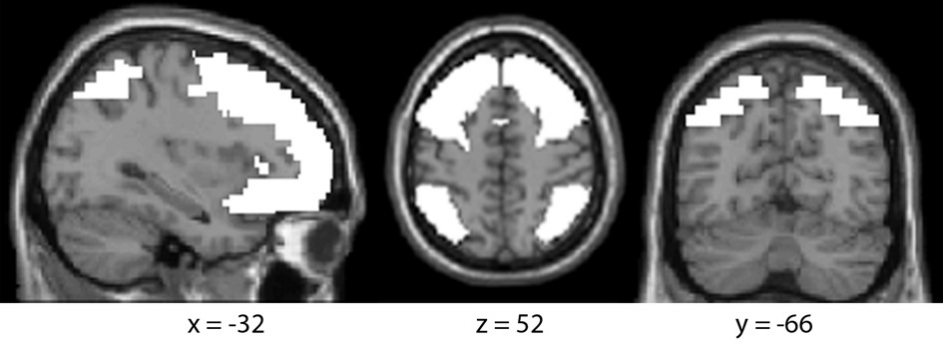
**Parietofrontal mask for fMRI analyses.** All fMRI analyses were performed within a mask of the frontal (IFG, MFG, SFG) and parietal (IPL, SPL) lobes.

***Second-level group statistics***. A randomized effects model was used for group analysis. All analyses were performed within a mask of frontal (IFG, MFG, SFG) and parietal (IPL, SPL) cortex (see Figure 2). This mask was created by combining the WFU pickatlas definitions of these regions, as implemented through the SPM8 toolbox. Comparisons between stimulus classes were first performed to examine differences in brain activity based on sign type. These results are reported at thresholds of peak voxel level *p* < 0.005 (uncorrected), cluster *p* < 0.05 (FDR corrected), cluster size *k* > 30.

Comparisons were then made examining areas that showed increasing activity in response to decreasing comparison distance (i.e., a typical distance effect), or in response to increasing distance (a reversed distance effect), as found with the behavioral distance effect analyses, for each comparison sign type. These analyses examined brain activity associated with the parametric modulator of distance for each sign type. The parametric modulator function looks for areas of the brain that show a significant relationship between the modulator (here, distance) and brain activity, beyond any activity variance accounted for by the main effects (here, comparison sign type). Distance was thus used as a continuous measure, not binned into distance categories. As such, this represents a further and more stringent analysis, as some variance has already been accounted for by sign type, and thus results are not as strongly significant as with the main effects. Given this difference, the results for activity related to the modulator of distance are reported at thresholds of peak voxel level *p* < 0.05, cluster size *k* > 10.

Co-ordinates are MNI using ICBM152. Anatomical regions were assigned by a combination of xjview (Cui et al., 2011), visual inspection, and Talairach daemon after transformation to Talairach space (Lancaster et al., 1997, 2000; Brett, 2006). The anatomical region listed is for the peak voxel location. In all cases, IPS activity was confirmed by hand if the analyses demonstrated significant activity peaks in the inferior or superior parietal lobules.

## Results

### Behavioral testing

Independent samples t-tests demonstrated that seventh-graders were significantly older than fifth-graders [*t*_(29)_ = 9.67, *p* < 0.001]. Neither KTEA-II standard scores [*t*_(29)_ = 1.301, *p* =0.204] nor Stroop interference standard scores [*t*_(27)_ = 0.522, *p* = 0.606] were significantly different between age groups, but seventh-graders performed significantly better than fifth-graders on the Integer Knowledge Test [*t*_(29)_ = 5.304, *p* < 0.001] (see Table 1 for group scores and means): no fifth-graders were able to perform multiplication or division with negative numbers, and all but three could not perform addition or subtraction, while all seventh-graders were at least able to perform addition and subtraction.

### Effects of sign and age

A 2 (sign) × 2 (age group) Repeated Measures ANOVA was first performed on accuracy data. Between subjects, there was no significant effect of age [*F*_(1, 29)_ = 1.71, *p* > 0.2, *MSE* = 0.019], indicating that fifth-grader (mean = 84.8%) and seventh-grader (mean = 88.3%) performance was similar on the task. Within subjects, there was a significant effect of sign, *F*_(1, 29)_ = 38.238, *p* < 0.001, *MSE* = 0.130, η^2^_*p*_ = 0.569, where responses to positive number comparisons (mean = 91.1%) were more accurate than to negative number comparisons (mean = 81.9%), but no significant sign × group interaction was found [*F*_(1, 29)_ = 3.166, *p* = 0.086, *MSE* = 0.011].

A 2 × 2 Repeated Measures ANOVA was then performed on response time data. Between subjects, there was a significant main effect of age, *F*_(1, 29)_ = 4.777, *MSE* = 221550, *p* = 0.037, η^2^_*p*_ = 0.141. Within subjects, there was a significant main effect of sign, *F*_(1, 29)_ = 98.822, *MSE* = 272424, *p* < 0.001, η^2^_*p*_ = 0.773, and a significant interaction between sign and age, *F*_(1, 29)_ = 8.247, *p* = 0.008, η^2^_*p*_ = 0.221. Seventh-grader responses (mean = 967.75 ms) were faster than fifth [mean = 1087.37 ms; *t*_(29)_ = 2.186, *p* = 0.037], and positive number comparison responses (mean = 961.24 ms) were faster than negative [mean = 1093.88; *t*_(30)_ = −9.009, *p* < 0.001]. Fifth- and seventh-grader response times did not differ for positive number comparisons [*t*_(29)_ = 1.527, *p* > 0.1], but seventh-grader responses were significantly faster than fifth for negative number comparisons [*t*_(30)_ = 2.664, *p* = 0.012].

### Distance effects

The presence and direction of a distance effect in participant response times was assessed using linear regressions. Response times for each comparison pair type were used as the dependent variable, and numeric comparison distance as the independent, to determine whether response time was significantly predicted by distance. Unstandardized beta coefficients for the distance regressor were extracted for each participant, once for the positive pair comparisons and once for the negative. The beta coefficients reflect the predicted change in response time (in milliseconds) for a one-unit change in distance; negative beta coefficients indicate a typical distance effect, as response times should decrease as distance increases, and positive beta coefficients a reversed distance effect. This analysis thus allows examination of the effect of distance on response time, but also removes the effect of overall response time differences between the sign categories. This analysis is the same as that used with adults (see Gullick et al., 2012). These beta-weights were then entered into one-sample *t*-tests to determine whether they were significantly different from zero, and thus showed a significant effect of distance on response times across individuals.

**Figure 3:**
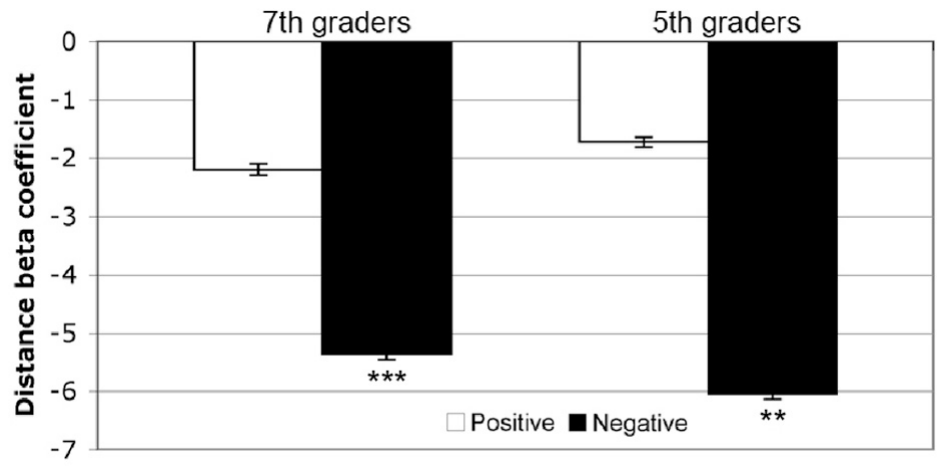
**Behavioral distance effects.** Both fifth- and seventh-graders demonstrated typical direction response time distance effects for negative number comparisons, but no significant effects for positive pairs. ^*^p < 0.05, ^**^p < 0.005, ^***^p < 0.001. Error bars indicate one standard error of the mean.

Fifth-graders demonstrated a typical distance effect for negative number comparisons [mean = −6.05, *t*_(15)_ = −3.565, *p* = 0.003], but no significant effect for positive number comparisons [mean = −1.72, *t*_(15)_ = −1.253, *p* = 0.229]. Seventh-graders demonstrated a typical distance effect for negative number comparisons [mean = −5.36, *t*_(14)_ = −5.238, *p* < 0.001], but no significant effect for positive number comparisons [mean = −2.19, *t*_(14)_ = −1.516, *p* = 0.152] (see Figure 3). These beta coefficients were then compared between age groups using a 2 (sign) × 2 (age group) Repeated Measures ANOVA. Between subjects, there was no significant main effect of age (*F* < 1). There was a significant main effect of sign, *F*_(1, 29)_ = 10.445, *MSE* = 217, *p* = 0.003, η^2^_*p*_ = 0.265, but no interaction between sign and age [*F* < 1]. Negative comparison pairs thus demonstrated a stronger distance effect than positive in each age group.

Positive number comparisons thus did not demonstrate a significant distance effect in these context-collapsed analyses, though negative number comparisons did. To further investigate this situation, we conducted separate analyses for each sign within each presentation context. Fifth-graders demonstrated a marginally significant typical distance effect for digit-context positive number comparisons [mean = −5.94, *t*_(15)_ = −2.063, *p* = 0.057], but not for negative numbers [mean = −2.42, *t*_(15)_ = −1.044, *p* > 0.3]. However, temperature-context comparisons showed a significant distance effect for negative number comparisons [mean = −11.11, *t*_(15)_ = −5.406, *p* < 0.001] but not positive [mean = 2.94, *t*_(15)_ = 1.700, *p* > 0.1]. Seventh-graders demonstrated significant distance effects for both signs in the digit context [positive number pairs: mean = −4.62, *t*_(14)_ = −3.158, *p* = 0.007; negative number pairs: mean = −3.59, *t*_(14)_ = −2.585, *p* = 0.022]. Similarly to the younger subjects, temperature-context comparisons showed a significant distance effect for negative number comparisons [mean = −7.79, *t*_(14)_ = −3.886, *p* = 0.002] but not positive [mean = 0.64, *t*_(14)_ = 0.316, *p* > 0.7]. As such, positive numbers always demonstrated a typical distance effect in digit-format presentations, but not when presented as thermometers, for both age groups. Negative numbers showed a significant distance effect when presented as thermometers in both groups, but a significant digit-format effect was found only for seventh graders. Despite these context-dependent outcomes, there were insufficient trials to separately analyze other potential digit- vs. thermometer-format trial effects. Thus, all further analyses collapse across presentation context, combining digit- and thermometer-format pairs within each sign.

### Predicting the size of the distance effect

We then investigated the factors predicting the size of the distance effect (beta coefficient) for negative number comparisons across individuals within each age group using stepwise linear regressions. In each regression, the negative number comparison beta coefficient was entered as the dependent variable, and negative comparison accuracy, negative response time, positive comparison accuracy, positive response time, positive comparison beta coefficient, age (in months), KTEA-II standardized score, Integer Knowledge Test score, and Stroop interference score (where applicable) were included as independent variables.

**Figure 4:**
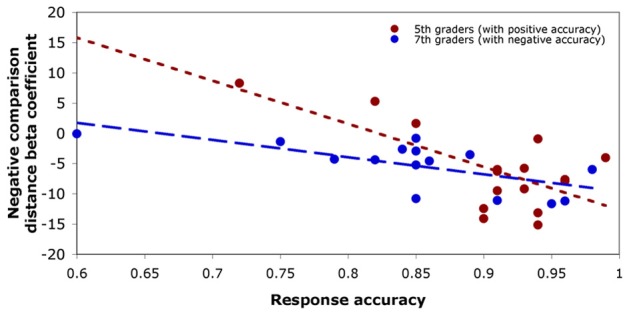
**Predicting the size of the negative number distance effect.** Fifth-graders (red) demonstrated a significant negative relationship between positive comparison accuracy and negative comparison distance effect. Seventh-graders (blue) demonstrated a significant negative relationship between negative comparison accuracy and negative comparison distance effect. In each case, participants with higher comparison accuracies showed a more negative (typical) distance effect.

In fifth-graders, only positive comparison accuracy significantly predicted the size of the negative number comparison distance effect, *t*_(14)_ = −3.45, *p* = 0.004. In seventh-graders, the size of the negative number comparison distance effect was predicted first by negative comparison accuracy, *t*_(14)_ = −3.24, *p* = 0.007, then additionally by Stroop interference score, *t*_(14)_ = 3.28, *p* = 0.007 (see Figure 4).

## fMRI results

### Effects of sign and age

**Figure 5:**
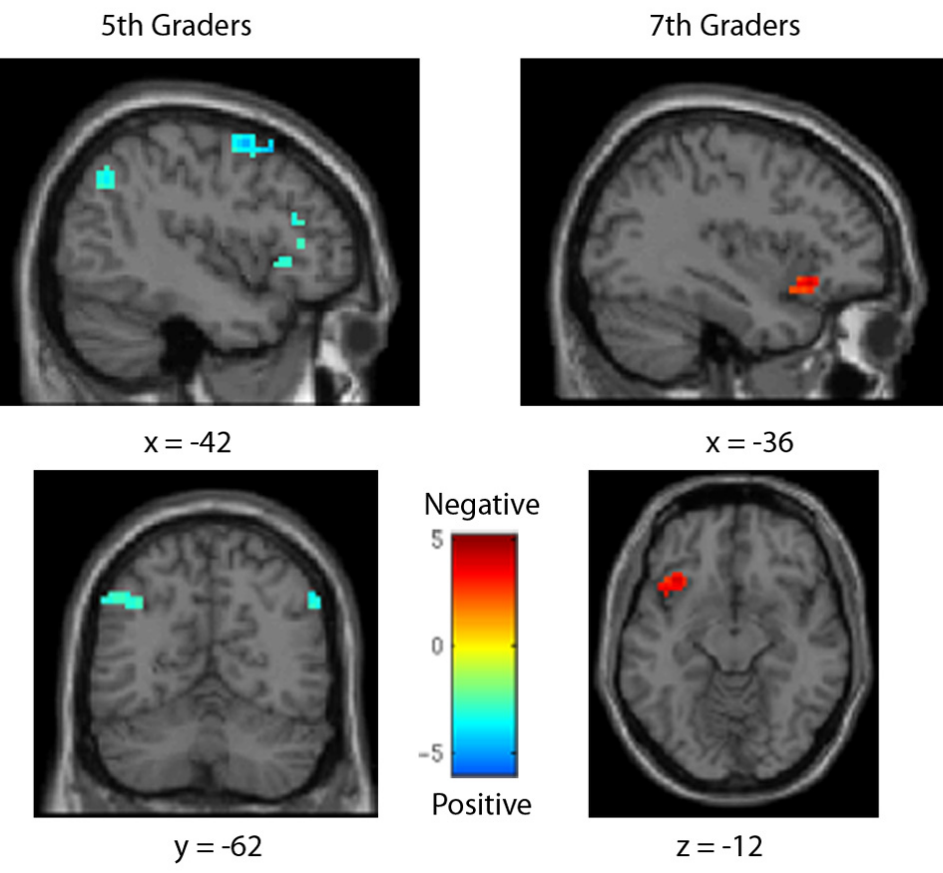
**Brain activity for positive vs. negative number comparisons in each age group.** For fifth-graders, positive number comparisons (cool colors) showed greater activity in the left IPS. For seventh graders, negative number comparisons (warm colors) demonstrated greater activity in only the left inferior frontal gyrus. *All fMRI figures show left-hemisphere activity on the left side. All figure colorbars indicate t-test contrast values*.

As with the behavioral analyses, data was first compared between sign types, across comparison distances, to determine areas differentially involved in processing positive and negative numbers within each age group (see Table 3, Figure 5). In fifth-graders, positive number comparisons showed greater activity than negative comparisons in the bilateral parietal lobe, including the IPS, as well as the bilateral inferior and superior frontal gyri; negative comparisons evoked more activity than positive in one cluster in the right inferior parietal lobule, though not in the IPS. In seventh-graders, negative comparisons evoked more activity than positive in one cluster in the left inferior frontal gyrus. No further differences were seen.

**Table 3:** **Positive versus negative comparisons, within each age group**.

**Location of peak voxel**	**MNI coordinates**			**Cluster size**	**Peak *t***
	***x***	***y***	***z***		
**FIFTH GRADERS POSITIVE > NEGATIVE NUMBERS**					
R inferior frontal gyrus	12	38	−20	89	5.92
R superior frontal gyrus	18	38	58	54	5.7
L superior, middle frontal gyrus	−21	38	58	148	5.61
L inferior frontal gyrus	−60	29	−2	121	5.6
L superior frontal gyrus	−12	−10	67	122	5.46
R inferior parietal lobule	42	−70	46	58	5.3
L superior frontal gyrus	−18	44	−17	101	4.97
R middle frontal gyrus	48	56	10	54	4.69
R inferior, middle frontal gyrus	60	14	31	214	4.51
R superior frontal gyrus	−9	62	1	68	4.01
L inferior parietal lobule	−42	−64	40	49	3.76
**FIFTH GRADERS NEGATIVE > POSITIVE NUMBERS**					
R inferior parietal lobule	42	−34	28	49	5.2
**SEVENTH GRADERS POSITIVE > NEGATIVE NUMBERS**					
None					
**SEVENTH GRADERS NEGATIVE > POSITIVE NUMBERS**					
L inferior frontal gyrus	−33	23	−11	47	4.01

### Distance effects

**Figure 6:**
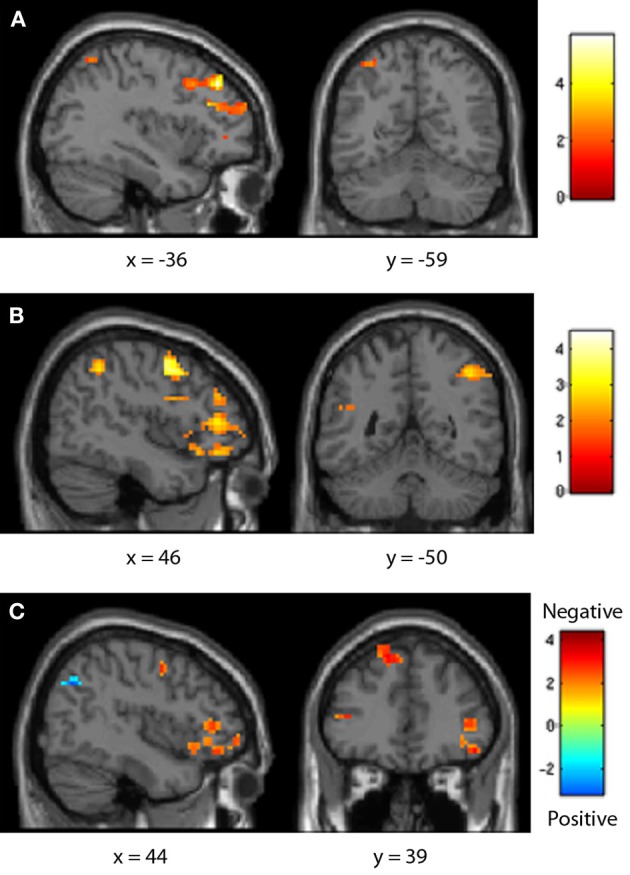
**Fifth-grader neural distance effects.** Both positive **(A)** and negative **(B)** number comparisons showed a typical distance effect in the frontal and parietal lobes, though for negative pairs this was only in the right IPS. This effect was greater for positive comparisons in the parietal lobe, but greater for negative pairs in the frontal lobe **(C)**.

fMRI data was then analyzed to determine brain areas showing a linear increase in activity as comparison distance increased, or decreased, within each sign category. Significant clusters were identified at a height threshold of *p* < 0.05 and cluster size of 10 voxels. In fifth-graders, positive comparisons demonstrated a typical distance effect both frontally (including the left inferior frontal and right precentral gyri) and parietally (including the left IPS). Negative comparisons also showed a typical distance effect across the frontal and parietal lobes, though in only the right IPS. This effect was greater for positive comparisons in the parietal lobe, including the right IPS, but was greater for negative comparisons in the frontal lobe (including the bilateral precentral and inferior frontal gyri) (see Table 4, Figure 6).

**Table 4:** **Fifth-grader neural distance effects for positive, negative number comparisons**.

**Location of peak voxel**	**MNI coordinates**			**Cluster size**	**Peak *t***
	***x***	***y***	***z***		
**FIFTH GRADERS POSITIVE DISTANCE EFFECT**					
L middle, inferior frontal gyrus	−36	35	40	372	5.69
R superior, middle frontal gyrus	24	50	1	615	4.6
L superior, middle frontal gyrus	−27	53	1	85	4.33
R superior frontal gyrus	9	26	61	26	2.75
R middle, inferior frontal gyrus	24	26	−20	23	2.71
R middle frontal gyrus	45	−1	61	18	2.68
L superior parietal lobule	−33	−58	58	11	2.59
L inferior frontal gyrus	−57	14	19	23	2.38
L inferior frontal gyrus	−42	44	1	27	2.38
**FIFTH GRADERS NEGATIVE DISTANCE EFFECT**					
Bilateral superior, right middle	0	14	55	304	4.51
frontal gyrus					
L superior frontal gyrus	−18	50	34	123	4.14
R inferior frontal gyrus	36	23	−11	141	3.93
R superior frontal gyrus	24	53	−11	75	3.67
L superior, middle frontal gyrus	−9	29	61	320	3.66
R superior frontal gyrus	6	8	67	54	3.33
R middle frontal gyrus	54	38	22	43	3.25
R inferior parietal lobule	51	−55	43	79	3.15
L inferior frontal gyrus	−48	−1	22	43	3.13
R inferior frontal gyrus	51	2	25	89	2.98
L middle, superior frontal gyrus	−24	59	−11	248	2.96
R inferior frontal gyrus	45	35	7	159	2.9
L superior frontal gyrus	−18	−19	70	73	2.75
R superior frontal gyrus	12	44	49	15	2.64
R middle, superior frontal gyrus	24	50	25	126	2.55
L inferior parietal lobule	−42	−43	22	22	2.45
R superior frontal gyrus	9	−19	73	10	2.39
L inferior frontal gyrus	−54	23	4	19	2.32
L inferior frontal gyrus	−48	29	16	15	2.16
**FIFTH GRADERS POSITIVE > NEGATIVE DISTANCE EFFECT**					
R inferior parietal lobule	45	−67	37	29	3.1
R inferior frontal gyrus	18	20	−20	21	2.79
R middle frontal gyrus	−36	56	25	11	2.56
R superior frontal gyrus	3	62	1	10	2.34
R superior frontal gyrus	24	65	22	11	2.07
**FIFTH GRADERS NEGATIVE > POSITIVE DISTANCE EFFECT**					
L middle frontal gyrus	−33	2	49	61	4.36
L superior frontal gyrus	−6	5	55	48	4.07
R inferior, middle frontal gyrus	51	44	−14	153	3.65
L inferior frontal gyrus	−39	20	−14	168	3.37
L superior frontal gyrus	−12	38	52	84	3.2
R middle frontal gyrus	24	−4	49	73	3.17
R superior frontal gyrus	15	17	49	16	3.01
L inferior frontal gyrus	−39	38	10	43	2.77
R inferior frontal gyrus	45	38	4	23	2.54
L superior frontal gyrus	−9	−4	70	13	2.52
R superior frontal gyrus	15	53	31	31	2.43
R superior frontal gyrus	12	−1	70	29	2.4
L inferior parietal lobule	−45	−37	46	24	2.33
R inferior frontal gyrus	60	11	28	10	2.22

**Figure 7:**
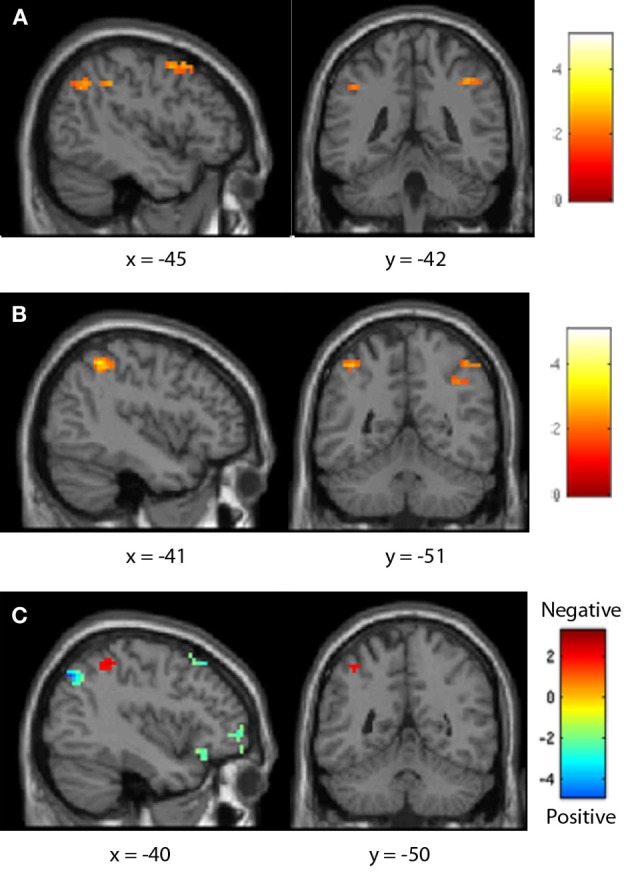
**Seventh-grader neural distance effects.** Both positive **(A)** and negative **(B)** comparisons demonstrated a typical distance effect in the parietal lobe. This effect was greater for positive comparisons in the right IPS and frontal lobe, but greater for negative pairs in the left IPS **(C)**.

In seventh-graders, positive comparisons demonstrated a typical distance effect both frontally (including the bilateral precentral gyrus and left inferior frontal gyrus) and parietally (including the bilateral IPS). Negative comparisons showed a typical distance effect in the parietal lobe (including the bilateral IPS). This effect was greater for negative comparisons in the left IPS, but was greater for positive pairs in the frontal lobe (including the bilateral inferior frontal gyrus and left precentral gyrus, see Table 5, Figure 7).

**Table 5:** **Seventh-grader neural distance effects for positive, negative comparisons**.

**Location of peak voxel**	**MNI coordinates**			**Cluster size**	**Peak *t***
	***x***	***y***	***z***		
**SEVENTH GRADERS POSITIVE DISTANCE EFFECT**					
R inferior frontal gyrus	12	38	−20	89	5.92
Bilateral superior frontal gyrus	15	−16	76	59	4.95
L middle, superior frontal gyrus	−39	29	52	122	4.15
L inferior frontal gyrus	−57	38	4	17	3.07
R middle frontal gyrus	45	−4	58	39	2.89
L superior, inferior parietal lobule	−36	−67	43	53	2.84
L inferior parietal lobule	−63	−34	34	32	2.76
R inferior parietal lobule	42	−43	46	27	2.47
L middle frontal gyrus	−42	56	4	28	2.45
L inferior parietal lobule	−45	−40	40	16	2.43
L inferior frontal gyrus	−33	26	−14	10	2.35
L middle frontal gyrus	−36	50	34	14	2.22
**SEVENTH GRADERS NEGATIVE DISTANCE EFFECT**					
R inferior parietal lobule	45	−49	28	38	3.37
L inferior parietal lobule	−63	−28	31	63	3.22
L inferior parietal lobule	−42	−49	52	60	3.21
R inferior parietal lobule	36	−34	40	56	3.08
R middle frontal gyrus	51	17	43	95	2.71
R middle frontal gyrus	30	−10	49	18	2.71
R superior frontal gyrus	18	17	58	14	2.65
R superior parietal lobule	39	−43	58	31	2.3
R superior parietal lobule	45	−52	52	11	2.22
**SEVENTH GRADERS POSITIVE > NEGATIVE DISTANCE EFFECT**					
L inferior, superior parietal lobule	−39	−73	43	77	4.84
L inferior frontal gyrus	−33	35	−17	245	4.16
L inferior frontal gyrus	−60	17	31	66	4.09
L middle, superior frontal gyrus	−48	5	55	201	4.01
R superior frontal gyrus	24	65	−8	20	2.94
R superior frontal gyrus	15	−16	79	10	2.78
R inferior frontal gyrus	57	38	−11	11	2.5
L superior frontal gyrus	−12	65	4	10	2.39
R inferior frontal gyrus	54	17	25	13	2.27
**SEVENTH GRADERS NEGATIVE > POSITIVE DISTANCE EFFECT**					
L inferior parietal lobule	−60	−28	28	17	3.29
R middle frontal gyrus	48	26	43	79	3.18
R inferior frontal gyrus	18	11	−20	11	2.72
R superior frontal gyrus	9	53	37	24	2.57
R inferior parietal lobule	−39	−43	52	18	2.22

**Figure 8:**
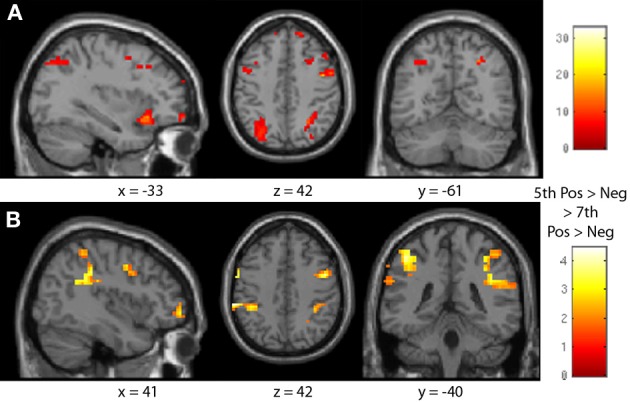
**Age by sign neural distance effect interaction. (A)** The bilateral intraparietal sulcus and parts of the inferior and middle frontal gyri were sensitive to the interaction of age group and comparison sign distance effects. **(B)** Fifth-graders showed a greater difference between positive and negative number distance effects in the bilateral IPS and bilateral inferior frontal gyrus than did seventh-graders.

The interaction between age group and the distance effect in each sign was then investigated using a 2 (sign) × 2 (age group) ANOVA (see Table 6, Figure 8). There were significant effects in the bilateral parietal lobule, including the IPS, as well as the bilateral frontal lobes, especially the inferior and middle frontal gyri (see Figure 8A). Differences in the distance effect in each sign between age groups were then investigated by comparing positive greater than negative number distance effects for fifth-greater than seventh-graders. Fifth-graders showed a greater difference between positive and negative number distance effects than seventh-graders in the bilateral IPS and inferior frontal gyrus (see Figure 8B).

**Table 6:** **Interaction between sign and age group on the distance effect**.

**Location of peak voxel**	**MNI coordinates**			**Cluster size**	**Peak *t***
	***x***	***y***	***z***		
**INTERACTION SIGN × AGE GROUP**					
R middle frontal gyrus	42	−4	49	128	32.9
R inferior, middle frontal gyrus	60	20	25	321	23.04
R middle, superior frontal gyrus	24	62	−11	19	20.29
L inferior, middle frontal gyrus	−48	14	−2	308	18.94
R inferior frontal gyrus	51	38	−14	45	17.41
R superior frontal gyrus	9	−19	76	76	16.88
R middle frontal gyrus	42	53	1	343	16.71
L superior frontal gyrus	−6	29	49	66	15.26
R superior, inferior parietal lobule	24	−64	46	62	15.13
R middle frontal gyrus	−24	8	49	78	14.35
R inferior frontal gyrus	−33	23	−11	67	14.31
Bilateral superior frontal gyrus	0	11	55	46	13.62
R inferior frontal gyrus	21	17	−23	45	13.4
R middle frontal gyrus	27	35	49	58	12.25
L inferior parietal lobule	−60	−28	34	17	12.16
R superior parietal lobule	33	−49	58	35	11.49
R inferior parietal lobule	39	−37	25	46	9.93
R superior frontal gyrus	12	62	28	14	9.93
R superior, inferior parietal lobule	−30	−70	43	65	9.53
R superior frontal gyrus	21	68	10	33	8.89
R middle frontal gyrus	33	20	37	48	6.13
L middle frontal gyrus	−39	17	40	13	5.57
**FIFTH > SEVENTH GRADERS POSITIVE > NEGATIVE NUMBER DISTANCE EFFECT**					
L inferior parietal lobule	−63	−34	43	282	4.44
Bilateral superior frontal gyrus	9	5	61	69	4.37
R superior frontal gyrus	24	−7	55	37	4.06
Bilateral superior frontal gyrus	−9	−1	73	129	3.92
R middle frontal gyrus	48	2	43	122	3.86
L middle, inferior frontal gyrus	−60	−1	46	41	3.64
R inferior parietal lobule	45	−49	28	341	3.64
L inferior parietal lobule	−36	−49	25	19	3.59
R inferior frontal gyrus	57	23	−11	14	3.34
R superior parietal lobule	24	−46	64	19	3.22
L superior parietal lobule	−18	−67	55	23	3.19
R superior parietal lobule	27	−64	52	28	3.08
R inferior frontal gyrus	42	56	−5	28	3.01
R inferior frontal gyrus	30	23	−23	20	2.89
L middle frontal gyrus	−33	−1	49	49	2.47
L inferior frontal gyrus	−39	11	−17	10	2.34
L inferior frontal gyrus	−45	17	−8	30	2.21

### Covariate effects on negative number neural distance effects

**Figure 9:**
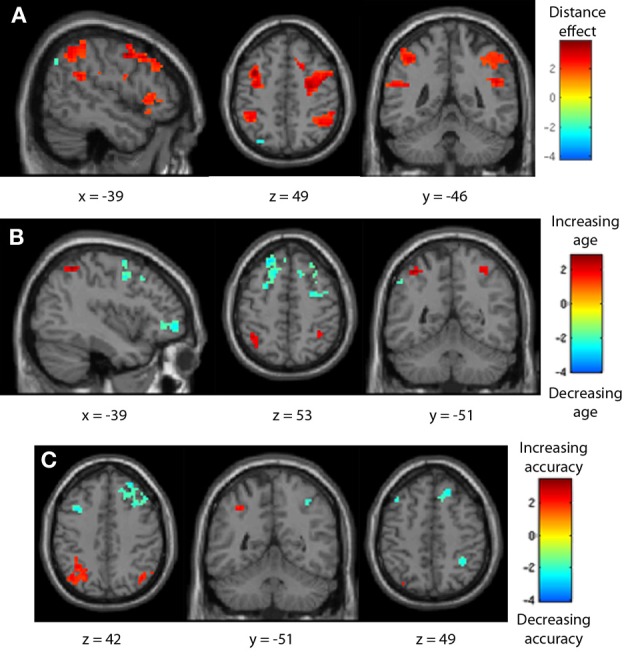
**Negative number neural distance effects across children.** Fifth- and seventh-graders together demonstrated a bilateral IPS distance effect for negative number comparisons. **(A)** A frontoparietal shift was seen with increasing age **(B)**, and a right-to-left laterality shift was seen in the IPS with increasing negative comparison accuracy **(C)**.

As comparison accuracy was found to be predictive of the size of the negative number distance effect behaviorally, these relationships were investigated neurally. The impacts of age and task accuracy on the negative number neural distance effect were assessed by including fifth- and seventh-grader negative number distance effect contrasts in the same regression analysis. Participant age (in months) and negative number comparison response accuracy were added as covariates of interest; age and accuracy were not significantly correlated (*r* = 0.299, *p* > 0.1), and so separable effects may be discussed. Across groups, there was a significant neural distance effect for negative number comparisons, with the bilateral IPS demonstrating increasing activity given decreasing comparison distance. As participant age increased, activity increased in the bilateral IPS and decreased in the bilateral inferior parietal lobule and bilateral inferior frontal gyrus and precentral gyrus. As task accuracy increased, activity increased in the left IPS, and decreased in the right IPS and bilateral caudate (see Table 7, Figure 9). As such, maturation may promote the frontoparietal shift previously noted, but increased task accuracy may either indicate or cause a laterality shift (right to left) within the parietal lobe.

**Table 7:** **Covariate effects on the negative number distance effect across children**.

**Location of peak voxel**	**MNI coordinates**			**Cluster size**	**Peak *t***
	***x***	***y***	***z***		
**NEGATIVE DISTANCE EFFECT ACROSS PARTICIPANTS**					
L middle frontal gyrus	−36	8	52	117	3.85
R middle frontal gyrus	27	−7	52	357	3.59
R middle, superior frontal gyrus	24	50	−11	90	3.34
R inferior parietal lobule	57	−52	46	182	3.2
R superior frontal gyrus	12	2	70	94	3.03
R inferior parietal lobule	48	−46	25	52	2.96
L inferior parietal lobule	−39	−46	49	82	2.79
R superior frontal gyrus	18	14	49	59	2.75
R inferior frontal gyrus	33	23	−11	163	2.75
L inferior parietal lobule	−42	−46	22	33	2.7
L inferior frontal gyrus	−48	−1	22	29	2.58
L superior frontal gyrus	−9	−19	70	23	2.57
L superior frontal gyrus	−6	−4	70	33	2.57
L superior frontal gyrus	−57	11	43	10	2.56
R superior frontal gyrus	15	53	34	42	2.54
R inferior parietal lobule	45	−25	28	10	2.36
L superior, middle frontal gyrus	−15	44	34	59	2.29
R inferior frontal gyrus	48	−1	25	14	2.25
L inferior frontal gyrus	−48	14	−2	24	2.1
**INCREASING ACTIVITY WITH INCREASING AGE**					
L superior parietal lobule	−12	−67	58	28	2.85
R middle frontal gyrus	36	8	37	24	2.72
R inferior parietal lobule	39	−40	34	23	2.72
L inferior, superior parietal lobule	−39	−52	55	46	2.53
R inferior parietal lobule	30	−43	58	35	2.28
R superior frontal gyrus	18	14	58	14	2.23
**DECREASING ACTIVITY WITH INCREASING AGE**					
L superior, middle frontal gyrus	−12	59	−11	273	3.97
R middle, superior frontal gyrus	21	65	−11	53	3.7
L middle, superior frontal gyrus	−27	8	49	273	3.37
R inferior frontal gyrus	51	41	−14	35	3.04
R inferior frontal gyrus	57	17	25	61	2.78
R superior, middle frontal gyrus	15	17	49	115	2.77
R middle frontal gyrus	24	−1	49	31	2.69
L superior frontal gyrus	−21	59	19	54	2.68
L inferior parietal lobule	−48	−37	22	25	2.66
L inferior parietal lobule	−54	−58	40	32	2.64
L middle frontal gyrus	−39	−1	61	16	2.44
L inferior frontal gyrus	−54	17	4	20	2.4
R inferior frontal gyrus	33	11	−20	12	2.36
L middle, superior frontal gyrus	−21	50	34	19	2.35
R middle frontal gyrus	48	56	−2	12	2.25
R inferior frontal gyrus	39	38	7	10	2.02
**INCREASING ACTIVITY WITH INCREASING NEGATIVE NUMBER ACCURACY**					
R superior frontal gyrus	18	65	−11	23	3.5
R inferior parietal lobule	54	−64	40	49	2.7
L middle frontal gyrus	−42	56	10	12	2.46
L middle frontal gyrus	−30	62	−14	18	2.44
R middle frontal gyrus	45	56	10	12	2.38
L inferior parietal lobule	−42	−67	43	72	2.34
**DECREASING ACTIVITY WITH INCREASING NEGATIVE NUMBER ACCURACY**					
L superior frontal gyrus	−18	50	4	61	4.04
R middle, superior frontal gyrus	51	38	−2	479	3.99
R middle, superior frontal gyrus	36	35	22	237	3.62
L inferior frontal gyrus	−57	41	7	19	3.07
R inferior parietal lobule	42	−40	34	55	3.02
R superior, middle frontal gyrus	21	32	46	108	2.99
L inferior, middle frontal gyrus	−24	29	−5	142	2.89
L middle frontal gyrus	−42	23	52	15	2.87
L superior frontal gyrus	−15	59	31	63	2.64
L middle frontal gyrus	−36	14	43	25	2.51
R inferior parietal lobule	39	−49	52	26	2.45
L inferior frontal gyrus	−30	11	−14	23	2.42
R inferior frontal gyrus	63	11	25	30	2.28
R superior frontal gyrus	24	−13	64	20	2.22
L superior frontal gyrus	−12	41	37	10	2.13

## Discussion

The goal of this study was to begin to describe the neural systems involved in negative number processing before, and soon after, formal instruction on the topic. Pre-instruction fifth-graders and post-instruction seventh-graders were included to examine the effects of age and knowledge level on negative number use. Examinations were primarily conducted between positive and negative number comparison effects to determine whether negative number processing used the same systems, in the same manner, as positives, in each age group.

### Positive versus negative number comparisons

Generally, children's behavioral performance was similar to that of the adults previously reported in Gullick et al. (2012): the pattern of positive responses being better (faster, more accurate) than negatives was conserved across ages. Pre-instruction fifth-graders showed reasonable task accuracies and response times for negative numbers, which may indicate some ability to sequence and work with negative numbers even before formal school experience.

The neural contrasts between positive and negative comparisons showed quite different effects across age groups. Seventh-graders showed similar activities for positive and negative comparisons across distances: negative pairs evoked more activity in the left inferior frontal gyrus and bilateral superior temporal gyri, but few other differences were seen, potentially indicating similar representations of the two signs after some instruction. Fifth-graders, though, showed significantly more parietal primary number area activity, including the IPS, for positive than for negative comparisons, and more frontal activity for negatives than positives. This reversed effect demonstrates that the parietal differences seen may not be due simply to difficulty discrepancies between the signs: negative comparisons were harder (slower, less accurate) for fifth-graders, and yet showed decreased parietal activity relative to the easier positive comparisons. Instead, fifth-graders may not yet treat negative numbers as fully “numeric” or quantitatively meaningful, thus limiting their activity in primary quantity-sensitive regions.

### Positive and negative number distance effects

Both age groups demonstrated typical-direction behavioral distance effects for negative number comparisons. Developmentally, the size of the distance effect decreases with age, and a smaller distance effect may indicate a more mature representation of number (Holloway and Ansari, 2008). The negative number distance effect in adults has been previously demonstrated to be larger than that for positive, demonstrating that even after years of practice the left side of the mental number line is not quite as mature as the right (Gullick et al., 2012). The increased effect seen in children may also support this idea, as these participants have received only limited instruction or informal practice with negatives.

Behaviorally, positive number comparisons did not demonstrate a significant distance effect in the context-collapsed analyses; however, context-specific analyses demonstrated a typical distance effect for digit-format comparisons. While thermometer-format presentations appeared to aid processing of negative numbers, especially for the relatively naïve fifth graders, they seemed to interfere with positive number processing for both age groups. Unfortunately, we did not have enough statistical power to compare fMRI data between presentation contexts, and instead collapsed across digit- and thermometer-format trials. As such, there may be interesting neural differences between thermometer and digit comparison processing, especially given the context by age effects on negative number behavioral distance effects, but we cannot investigate these questions here. Further work is needed to better understand the impact of presentation context on number processing for both already-known positive numbers and recently-introduced negative numbers.

Neurally, all age groups demonstrated a typical distance effect for positive comparisons in the IPS, as well as in the precentral and inferior frontal gyrus. Previously, Ansari et al. (2005) noted only a right-lateralized frontal distance effect for children ages 8–12; while parietal regions were sensitive to number, their activity did not significantly differ between close and far comparison distances. Distance effects were proposed to shift at some point from the child-like frontal regions to the adult-like parietal regions, but the timeline of this shift was not more specifically defined. The children included in the current study fall in the upper end of this age range, and did show a parietal distance effect, as well as a right-lateralized precentral gyrus effect similar to that noted by Ansari et al. (2005) and a left-lateralized inferior frontal gyrus effect. Further, Ansari et al. (2005) categorized comparison distance as “close” or “far” and contrasted the two, whereas the present study used distance as a continuous parametric regressor, which may be more sensitive to small but important changes in processing. This study thus helps to better track the developmental trajectory of the frontoparietal shift even for positive number usage.

Similarly, both age groups demonstrated a typical distance effect for negative comparisons in the IPS and the precentral and inferior frontal gyri. Negative numbers thus draw on quantity-sensitive regions in a manner similar to that for positive comparisons, even in pre-instruction children. However, positive number comparisons demonstrated a greater distance effect in the IPS than negative comparisons in pre-instruction children; post-instruction children showed fewer neural differences between positive and negative number distance effects. As such, fifth-graders showed a greater difference between distance effects in each sign than did seventh-graders. These results may again indicate that while negative numbers may use primary number regions, it is not to the same degree as positives, at least before formal instruction and practice have occurred. This difference may again imply a less mature representation of negative numbers' quantities before instruction.

### Individual variability in the negative number distance effect

While on average fifth-grade children demonstrated a particularly immature distance effect for negative numbers, there was a large amount of individual variability within the group: some children did not show any consistent effect of distance on negative comparison response time, and some showed a large effect. Though a larger distance effect may indicate relative immaturity of or decreased precision in numeric representations, no distance effect for negative number comparisons at all may more indicate that negatives are not ordered or sequentially arranged. Fifth-graders' negative number distance effect was predicted only by their accuracy on positive number comparisons: children with low positive comparison accuracy tended to show a very small or reversed distance effect for negatives (implying a lack of organization), but higher-accuracy children were more likely to show a typical-direction distance effect. More broadly, children who were better at working with numbers generally were better at working with negative numbers specifically. A better understanding of quantity overall may provide a stronger base from which to work with difficult concepts like negative numbers, leading to a more mature effect. This finding suggests that, before instruction on this difficult concept, use of negatives may rely on one's ability to work with positives.

In contrast, seventh-graders' negative number comparison distance effect was predicted by negative comparison accuracy and Stroop interference score. First, children with higher negative comparison accuracies were more likely to show a typical distance effect for negative numbers. After instruction on negative numbers, then, the ability to use negatives may be based more on one's understanding of negatives themselves, and not on positive numbers. This difference between ages may represent a shift in understanding, though the mechanism behind this change cannot yet be discerned.

Second, the impact of the Stroop interference score on seventh-grader negative number distance effects may indicate the inhibition necessary in responding to negative numbers: to choose the greater number, participants must pick the smaller digit, which may be especially difficult in closer comparisons with a smaller difference between the values. However, the restriction of this effect to only the seventh-graders may indicate the prerequisite of some knowledge of negatives for inhibition to differentially effect responses across comparison distances.

As this study is cross-sectional, and occurred in a specific area where negative numbers are taught in the same grade across schools, it necessarily confounds participant age with instruction. Only the older seventh-grade children had learned about negative numbers in school, while none of the younger fifth-graders had. Luckily, the experimental sample included a range of ages and abilities within each grade group, making it possible to separately examine the impact of these two factors on brain activity and negative number performance. Both groups together demonstrated an expected parietal (IPS) and frontal (inferior frontal gyrus, precentral gyrus) distance effect for negative number comparisons. Changes in participant age were associated with the frontoparietal shift previously noted: negative number frontal distance effects decreased and parietal distance effects increased as participant age increased. This finding may again reflect the general developmental trend of a frontoparietal shift in numerical cognition (Ansari et al., 2005; Rivera et al., 2005). Interestingly, Rosenberg-Lee et al. (2011) reported a non-linear increase in both frontal and parietal activity in children from second to third grade in arithmetic problem solving, perhaps similar to the non-linear shifts seen here.

Alternately, the increased frontal distance effect for fifth-graders may be the result of relatively heightened strategy implementation. Pre-instruction children may primarily understand negatives through the use of rules, such as sign changes, which transform unfamiliar negative numbers into known positive values (see Varma and Schwartz, 2011); these rules may be especially important in solving close-distance pairs. With further instruction and experience, though, these rules may become less necessary, giving rise to the age-related differences seen.

Changes in participant accuracy on negative number comparisons, though, were associated with a right- to left-hemisphere IPS change. As participants' ability to work with negative numbers and to treat them as quantitative values increases, the distance effect for negatives seems to increase in the left IPS and decrease in the right. This pattern is also seen between age groups in the transition from a right IPS only negative number distance effect in fifth-graders to the bilateral effect seen in seventh-graders. This laterality shift may align with previously proposed separations within the mental number system. The right IPS has been noted to be more responsive to non-symbolic than symbolic number representations, and has been hypothesized to particularly support approximate quantitative processing. The left IPS, in contrast, is responsive to quantitative information across notations but is especially sensitive to practiced, enculturated symbolic numbers (Ansari, 2007; Kadosh et al., 2007; Piazza et al., 2007) and thus may be more able to represent exact amounts via symbolic cognition.

Within this comparison task, then, low performers may be more likely to understand negative numbers approximately, representing them as approximately small values via the right IPS, and thus also demonstrating larger behavioral distance effects. High-performers (and adults) may be more able to represent negative numbers as precise values, thus demonstrating increased distance-related activity in the left IPS and more mature distance effects. Whether the ability to represent negative values precisely causes or follows the shift cannot be determined from this study, but it does at least appear to be related.

## Summary

This study thus presents a first examination of the neural correlates of negative number processing in pre- and post-instruction children. As younger participants were expected to demonstrate a more frontal-based distance effect, and older possibly a more parietal-focused effect, neural analyses were first conducted separately within each age group to better directly compare the activities found in positive and negative pairs. Pre-instruction fifth-grader responses were, on average, reasonably accurate, demonstrating some knowledge of negative numbers even before formal instruction, and showed similar behavioral response patterns to older participants, indicating typical number line arrangement and use. All participants demonstrated a significant neural distance effect for both positive and negative number comparisons in the IPS, though frontal effects were also seen. Even before instruction, then, children may be able to treat negative numbers as representations of quantitative values and draw on number-related brain regions, even if in a relatively immature manner. Increasing age demonstrated a significant frontoparietal shift consistent with previous developmental numerical cognition work, but increasing negative comparison accuracy showed a right IPS to left IPS shift, possibly indicating a change from approximate to precise negative quantity representations. These shifts and changes illustrate the process of incorporation of negative numbers as quantitative entities into the mental number system across years of practice and experience.

### Conflict of interest statement

The authors declare that the research was conducted in the absence of any commercial or financial relationships that could be construed as a potential conflict of interest.

## Appendix

Integer Knowledge Test.

Write these numbers with the opposite sign:

**Table:**

1. −7 ________________________	2. 11 ________________________	3. 137 ________________________
4. 4 ________________________	5. −22 ________________________	6. −13 ________________________

Place these numbers on a number line:

**Table:**

1. 4	3. −2
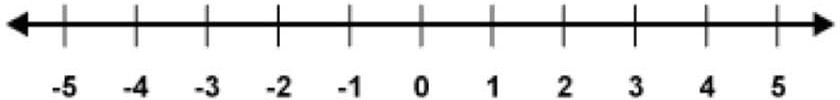
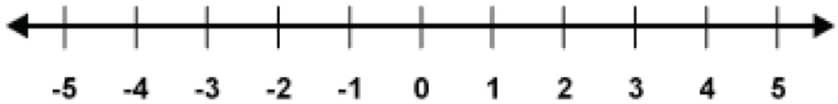
2. 1	4. −5
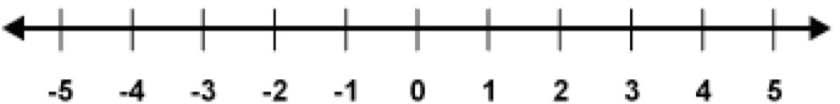
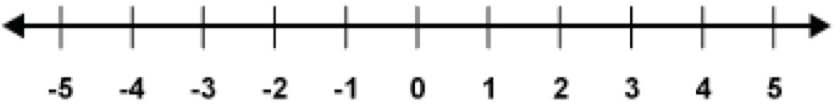

Order these numbers from least to greatest:

**Table:**

1. 0, −4, 4 ________________________________________	2. −2, −5, 6, 1 ______________________________________
3. −3, 24, 17, −6, 2, 1 _________________________________________________________________________________	
4. 19, 17, −17, −19, 0 _________________________________________________________________________________	

Circle the greater number:

**Table:**

1. 5 or 9	2. −3 or −8	3. −19 or −12
4. −2 or 6	5. 17 or −14	6. 16 or −21

Add the following integers:

**Table:**

1. 13 + 1 = ____________________________________	2. −4 + −6 = _________________________________
3. −2 + −12 = _________________________________	4. −6 + 8 = __________________________________
5. −11 + 8 = ___________________________________	6. 2 + −11 = _________________________________

Subtract the following integers:

**Table:**

1. 7 − 3 = _____________________________________	2. −10 – 4 = _________________________________
3. 5 − 2 = _____________________________________	4. −12 – −5 = _______________________________
5. −6 −3 = ___________________________________	6.4 – −9 = __________________________________

Multiply these integers:

**Table:**

1. 6 × 7 = ____________________________________	2. −4 × −9 = _________________________________
3. −8 × −3 = _________________________________	4. −10 × 5 = _________________________________
5. 6 × −9 = ___________________________________	6. −2 × 7 = __________________________________

Divide these integers:

**Table:**

1. 36 ÷ 2 = ___________________________________	2. −12 ÷ −4 = _______________________________
3. −45 ÷ −5 = ________________________________	4. −18 ÷ 6 = _________________________________
5. 24 ÷ −3 = __________________________________	6. −90 ÷ 10 = ________________________________

Solve for *x* in each case:

**Table:**

1. *x* − 3 = 12 _________________________________	2. 48 − x = 31 _______________________________
3. *x* ÷ −11 = 2 ________________________________	4. *x*/−5 = −8 _______________________________
5. *x* + 8 > 18 _________________________________	6. *x* − 1 < 3 _________________________________
7. *x* + 8 > −4 _________________________________	8. *x* ÷ 4 < 3 _________________________________
9. −5x > 25 __________________________________	10. −6x < −42 _______________________________
11. −5 < x/4 __________________________________	12. *x*/4 < −1/4 _______________________________

Write an equation describing each of these word problems, then solve it:

1. Maggie owes the candy store $35. Five of her friends will help her pay off her debt. How much will each friend pay? (Maggie has no money to help pay).
2. The temperature at noon on a winter day was 8°C. At midnight, the temperature had dropped by 15°. What was the temperature at midnight?
3. A drawing of a building shows the elevation of the basement floor to be 12 feet below ground level (–12 feet). The elevation of the roof is 32 feet. What is the total distance from the roof to the basement?
